# CANPMR syndrome and chromosome 1p32-p31 deletion syndrome coexist in two related individuals affected by simultaneous haplo-insufficiency of *CAMTA1* and *NIFA* genes

**DOI:** 10.1186/s13039-016-0219-y

**Published:** 2016-02-03

**Authors:** Emanuele G. Coci, Udo Koehler, Thomas Liehr, Armin Stelzner, Christian Fink, Hendrik Langen, Joachim Riedel

**Affiliations:** Center of Social Pediatrics and Pediatric Neurology, General Hospital of Celle, 29221 Celle, Germany; Medizinisch Genetisches Zentrum, 80335 Munich, Germany; Institute of Human Genetics, Friedrich Schiller University, Jena University Hospital, 07743 Jena, Germany; Department of Radiology, General Hospital of Celle, 29223 Celle, Germany

**Keywords:** NFIA, Chromosome 1p32-p31 deletion syndrome, CAMTA1, CANPMR syndrome, Paracentric inversion on short arm of chromosome 1

## Abstract

**Background:**

Non-progressive cerebellar ataxia with mental retardation (CANPMR, OMIM 614756) and chromosome 1p32-p31 deletion syndrome (OMIM 613735) are two very rare inherited disorders, which are caused by mono-allelic deficiency (haplo-insufficiency) of calmodulin-binding transcription activator 1 (*CAMTA1*) and, respectively, nuclear factor 1 A (*NFIA*) genes. The yet reported patients affected by mono-allelic *CAMTA1* dysfunction presented with neonatal hypotonia, delayed and ataxic gait, cerebellar atrophy, psychological delay and speech impairment, while individuals carrying a disrupted *NFIA* allele suffered from agenesis/hypoplasia of the corpus callosum, ventriculomegaly, developmental delay and urinary tract abnormalities. Both disorders were not seen in one patient together before.

**Results:**

In this study two related individuals affected by a complex clinical syndrome, characterized by cognitive, neurological and nephrological features were studied for the underlying genetic disorder(s) by molecular cytogenetics. The two individuals present dysmorphic facies, macrocephaly, generalized ataxia, mild tremor, strabismus, mild mental retardation and kidney hypoplasia. Moreover, neuro-radiological studies showed hypoplasia of corpus callosum. Genetic investigations revealed a paracentric inversion in the short arm of one chromosome 1 with breakpoints within *CAMTA1* and *NFIA* coding sequences.

**Conclusions:**

To the best of our knowledge, this is the first report of two patients harboring the simultaneous mono-allelic disruptions and consequent haplo-insufficiencies of two genes due to an inversion event. Disruption of *CAMTA1* and *NFIA* genes led to neuro-psychological and nephrological dysfunctions, which comprised clinical features of CANPMR syndrome as well as chromosome 1p32-p31 deletion syndrome.

## Background

Non-progressive cerebellar ataxia with mental retardation (CANPMR, OMIM 614756) is a very rare neuro-developmental disorder, belonging to the heterogeneous family of genetically determined cerebellar ataxias [1, 2] with recessive [3] and dominant [4] pattern of inheritance. The affected patients present with ataxic gait, dysmetries, variable mental retardation, cerebellar abnormalities and dysmorphic facies with heterogeneous penetrance. To date few genes/loci have been associated to autosomal recessive forms of cerebellar ataxias: *ATCAY* [5], chromosome 20q11-q13 locus [6], *VLDLR* [7], *ZNF592* [8], *SPTBN2* [9], *CWF19L1* [10], *PMPCA* [11]. Calmodulin-binding transcription activator 1 (*CAMTA1*) maps on chromosome 1p36, carries 23 exons and encodes 2 protein isoforms in mammalians. The brain-specific transcription factor CAMTA1 functions as homodimeric complex binding to gene promoters’ CGCG box thorough CG-1 domain, supporting the assembly of other transcription factors (e.g. Nkx2-5) and enhancing transcription of effector genes (e.g. *Fbxl4*) [12–14]. *CAMTA1* dysfunction has been associated to human pathology by Thevenon et al. [15], who reported five patients affected by *CAMTA1* haploinsufficiency due to deletions or duplications in the gene region coding for CG-1 domain, which plays a pivotal role in the whole CAMTA1 function. As mentioned above, the reported patients suffered from ataxia, broad-based gait, tremor, intellectual impairment and speech delay, cerebellar abnormalities (atrophy of lobes and/or vermis) and facial dysmorphisms (strabismus, large forehead, wide and broad nose, small ears).

The clinical features of chromosome 1p32-p31 deletion syndrome (OMIM 613735) were firstly described by Campbell et al. [16]. Some years later, Lu et al. proposed the causal association between this malformation syndrome and Nuclear Factor 1 A (*NFIA*) haplo-insufficiency [17]. *NFIA* maps on 1p31.2, carries 11 exons and produces at least 9 different protein isoforms [18–20]. The protein is functionally divided in two sections: a 200 amino acid long N-terminal DNA binding and dimerization domain, mainly encoded by exon 2, and C-terminal transactivation and repression domains, mainly encoded by exons 3 to 11. The first one binds to the nucleotide consensus sequence TTGGC(N)_5_GCCAA within the promoter region of several genes. The latter ones operate by directly activating basal transcription factors at transcription start sites, by displacing repressive histones from target genes, by interacting with other co-activator proteins [20]. All five individuals reported by Lu et al. (three of which previously described by Campbell et al.) presented with hypoplastic or absent corpus callosum, ventriculomegaly with or without relevant hydrocephalus and development delay; some of them carried urinary tract abnormalities (3 patients), epileptic seizures (3 patients), tethered spinal cord (4 patients) and Chiari malformation (3 patients). Although the chromosomal abnormalities [a translocation t(1;20), a translocation t(1;2), a 2.2 Mb deletion in 1p31-p32, a 12 Mb deletion in 1p31-p32] differed and the deleted regions comprised different genes among the five patients, only *NFIA* gene was either disrupted or fully deleted in all five patients, thus underpinning the association between *NFIA* haplo-insufficiency and the common CNS abnormalities (hypoplasia of corpus callosum, ventriculomegaly and hydrocephalus). A strong confirmation on their pathophysiological hypothesis was given by the clinical and histopathological findings of *Nfia*^-/-^ mouse model [17, 21]. A detailed study of the genome expression profile in murine *Nfia*^-/-^ brain at embryonic and post-natal stages showed a very strong imbalance in time-related expression of several genes playing a pivotal role in oligodendrocyte differentiation (e.g. *Mag, Mal, Mobp, Mog, Sox2, Sox4, Sox11, Dio2, Myef2*) as well as in axonal growth/guidance (e.g. *Clusterin, aFGF, Ndrg2, EphrinB2, Crmp1*) [22]. The essential role of transcription factor Nfib and Nfic in brain, tooth and lung development was already described in the corresponding mouse models [23, 24]. Few further reports described heterogeneous clinical findings associated with deletions mapping on chromosome 1p31 and 1p32 and encompassing several other genes [25, 26].

**Fig. 1:**
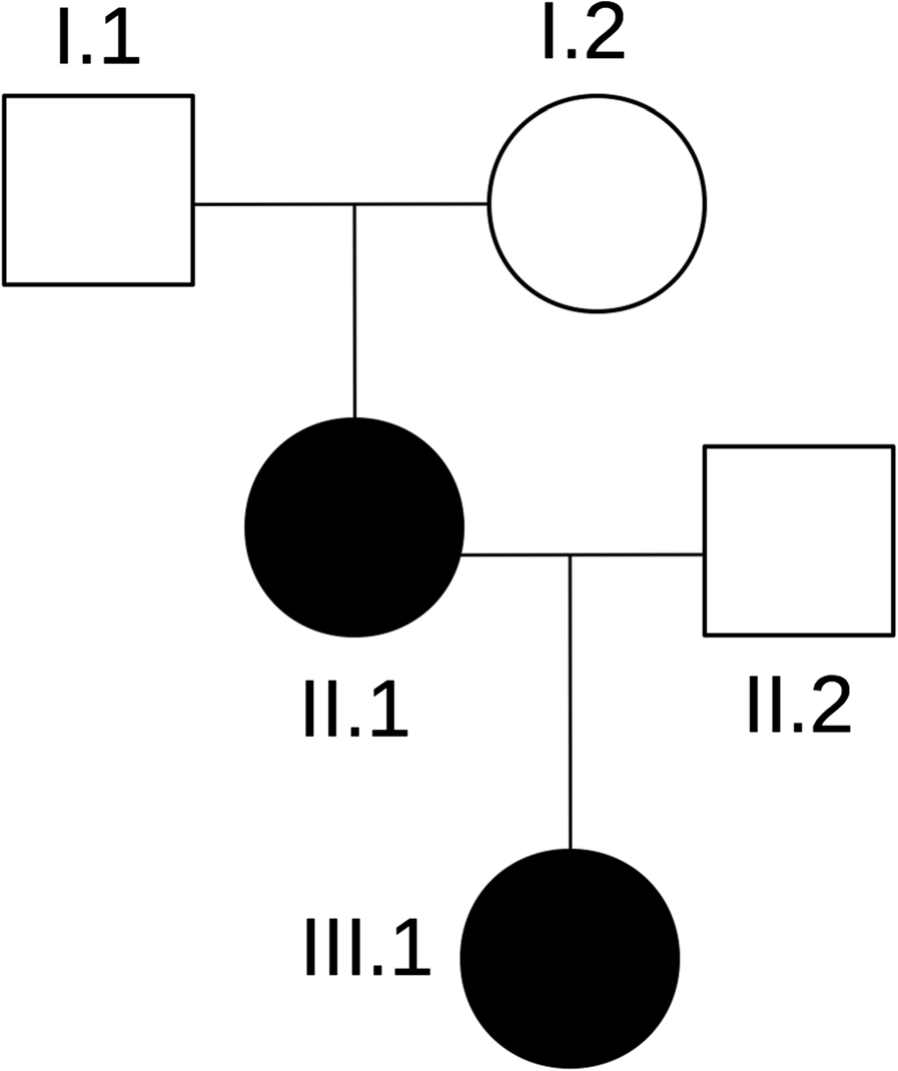
Pedigree of the affected family. The index-patient (III.1) and her mother (II.1) presents with the same karyotype

We describe the first family (Fig. 1) with two related individuals (II.1 and III.1) carrying simultaneous disruptions and consequent haplo-insufficiencies of *NFIA* and *CAMTA* genes due to a paracentric inversion in the short arm of chromosome 1 and presenting with clinical signs of CANPMR syndrome as well as chromosome 1p32-p31 deletion syndrome.

## Results and discussion

### Patient 1

Since the first months of life the index-patient (III.1) presented with generalized hypotonia, reduced muscular strength, particularly evident at trunk und pelvis, normal tendon reflexes, large head (> P90), prominent forehead, strabismus divergens and light bilateral ptosis. The patient could sit at 11 months and walk at 23 months of age. Gowers’ sign was through-out positive. Light dyskinesias and ataxia appeared at 3 years and, respectively, 6 years of age. She could speak the first words at about 30 months of age.

She slowly improved her motor, cognitive and language skills. Now, at the age of 7 years she can walk unassisted, climb stairs and speak short sentences. Her daily skills are compromised and she attends a special care kindergarten. Her intelligence quotient (IQ) score of 51 (Snijders-Oomen Non-Verbal test, SON-R) indicates a severe intellectual impairment (Table 1).

**Table 1:** Clinical, psychological, radiological features of the affected patients II.1 and III.1

	II.1 (28 years)	III.1 (6 ½ years)
Development parameters		
Sitting age (months)	8	11
Walking age (months)	14	23
Speaking age (months)	12	30
Clinical findings		
Macrocephaly	Yes (P 97)	No (P 90)
Muscle tonus	Normal	Normal
Seizures	No	No
Facies		
Forehead	Large	Large
Strabismus	Yes (divergens)	Yes (divergens)
Nasal bridge	Broad	Broad
Ears form and position	Normal	Normal
Mouth form and occlusion	Normal	Normal
Eye distance	2.5 cm (intercantal) and 6 cm (interpupillar)	2.5 cm (intercantal) and 5.5 cm (interpupillar)
Cerebellar symptoms		
Ataxic gait	Yes	Yes
Instability	Yes	Yes
Dysmetry	Yes	Yes
Dysartria	Yes	Yes
SARA score	6/40	11/40
Kidney and urinary tract defects	Recurrent infections, hypoplasia of the right kidney	Recurrent infections
Intelligence quotient (SON-R scale)	65	51
Brain MRI findings		
Corpus callosum hypoplasia	Hypoplastic	Hypoplastic
Ventriculomegaly or hydrocephalus	No	No
Cerebellar abnormalities	No	No

*SARA* Scale for the Assessment and Rating of Ataxia

**Fig. 2:**
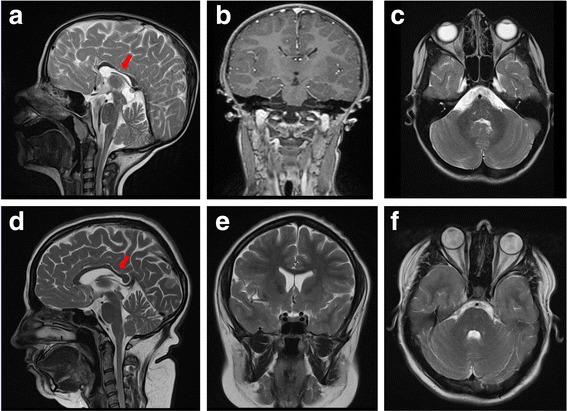
Brain MR investigation of index-patient (III.1, pictures **a**, **b**, **c**) and her mother (II.1, pictures **d**, **e**, **f**). Hypoplasia of the corpus callosum is revealed in both patients (red arrows). Otherwise no other structural anomalies were observed

The electroencephalogram (EEG) did not show epileptic discharges. The brain MR investigations at 2 and 6 ½ years of age revealed hypoplasia of corpus callosum, while the cerebellum was structurally normal (Fig. 2). Organ ultrasound did not show any structural abnormalities of kidneys and urinary tract, although the patient suffered from recurrent urine infections starting from 18 months of age.

### Patient 2 and other individuals of pedigree

The mother (II.1) of the index patient (28 years) presents with a similar clinical constellation of III.1, whereby she suffered from neonatal hypotonia, but sat unassisted at 8 months, walked at 14 months and spoke the first words at 12 months of age. She presents with a moderate ataxia, gait instability and dysmetria, which does not strongly impair the daily skills. Her IQ score of 65 (SON-R test) revealed a mental retardation. She has not completed any job training course and is currently unemployed. The brain MR scan, performed at 28 years of age, revealed hypoplasia of corpus callosum (Fig. 2).

Since the early childhood, she suffered from recurrent urinary tract infections due to vesicoureteral reflux (VUR) and from hypoplasia of the right kidney. The father (II.2) of the index patient carries a normal male karyotype and is a healthy individual. The maternal grandparents (I.1 and I.2) are healthy individuals; nevertheless their karyotype could not be analyzed due to missing compliance.

### Cytogenetic investigations

**Fig. 3:**
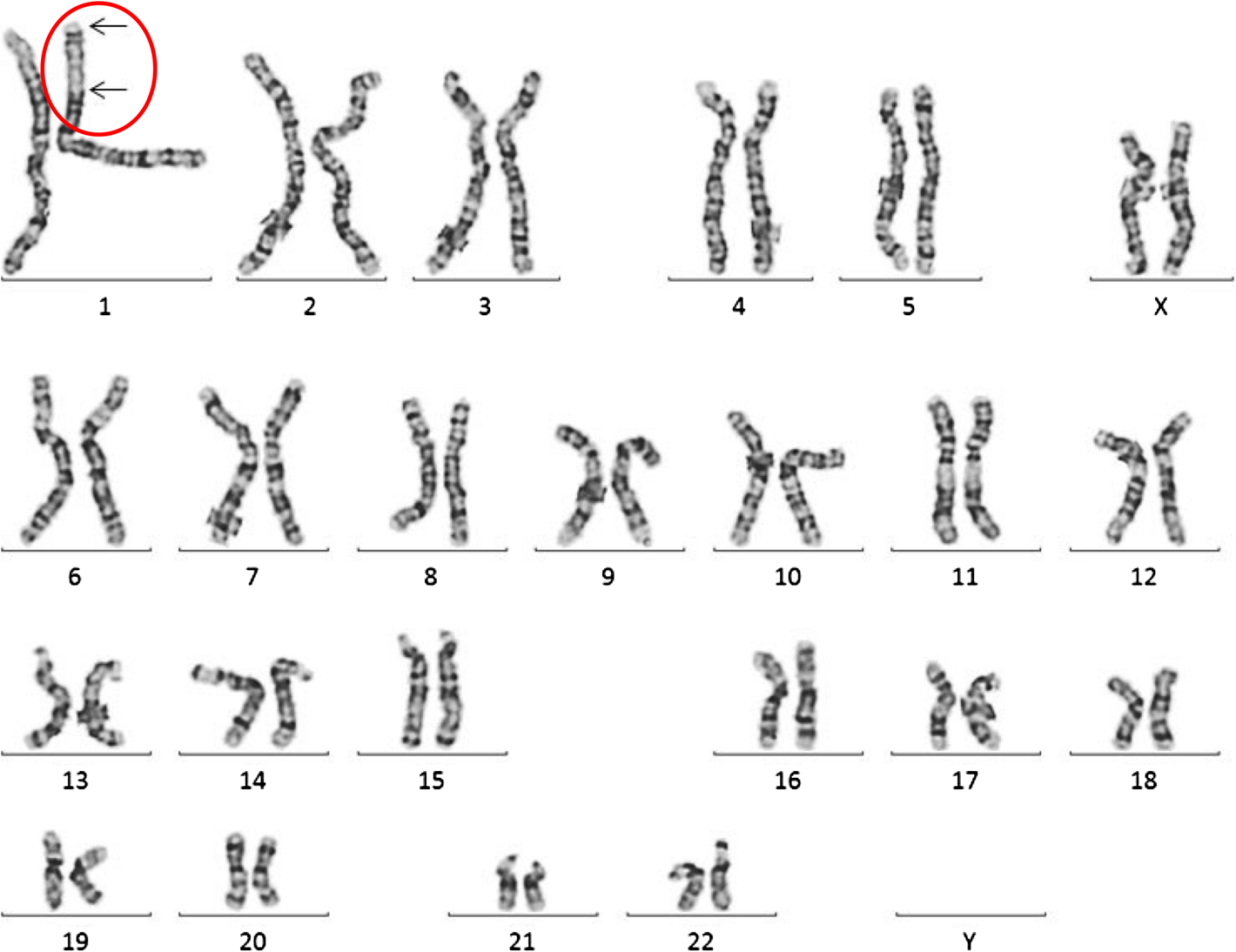
Karyotype of index-patient (III.1). Karyotyping (GTG-banding) of lymphocytes of peripheral blood revealed an inversion in the short arm of chromosome 1. Breakpoints in 1p31.3 and 1p36.31 (arrows) correspond to two deletions, which have been revealed by SNP array and depicted in Fig. 4

The index-patient III.1 harbors a paracentric inversion in the short arm of chromosome 1 with breakpoints within *CAMTA1* (1p36.31) and *NFIA* (1p31.3) genes (Fig. 3).

**Fig. 4:**
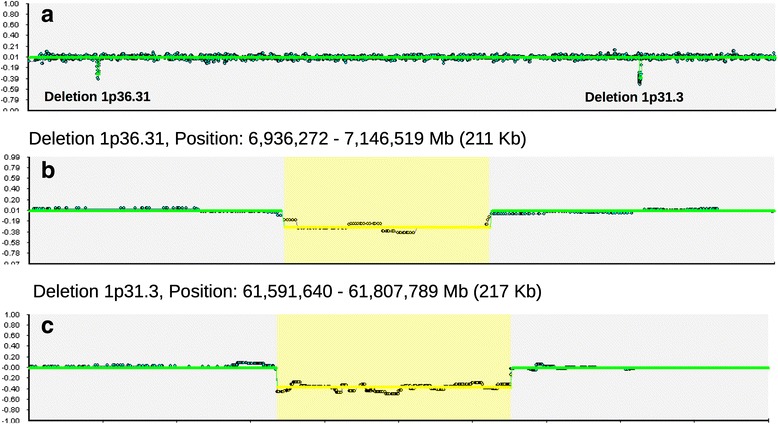
SNP Array on DNA from peripheral blood leukocytes. **a** Two deletions are revealed in the short arm of chromosome 1 in both the index-patient (III.1) and her mother (II.1). The distal deletion maps in band 1p36.31 and spans over 211 Kb (**b**) and the proximal deletion maps in band 1p31.3 and spans over 271 Kb (**c**). (GRC37h/hg19 genome build)

In correspondence of both breakpoints, two deletions occurred affecting *CAMTA1* exon 5 (deletion length 211 Kb; position 6,936,272 - 7,146,519 Mb, GRCh37/hg19) and *NFIA* exons 3-4 (deletion length 217 Kb; position 61,591,640 - 61,807,789 Mb, GRCh37/hg19) in patients II.1 and III1, which could only be resolved by SNP array (Fig. 4).

The resulting genotype is described as [46,XX,inv(1)(p36.31p31.3).arr 1p36.31(6,936,272-7,146,519)x1mat, 1p31.3(61,591,640-61,807,789)x1mat]. Due to the chromosomal inversion, the remaining coding regions of the affected alleles are restricted to exons 1 to 4 for *CAMTA1* and, respectively, exons 1 to 2 for *NFIA*. For *CAMTA1*, C-terminal 2/3 of CG-1 domain is deleted (about 80 out of 120 amino acids) and the other 3 C-terminal domains (TIG, IQ motif and Ankirin) are fully deleted. For *NFIA*, the N-terminal DNA binding domain (encoded by exon 2) is not affected by the deletion, but the C-terminal transactivation and repression domains (including also the proline-rich domain) are absent from the remaining coding sequence. The second affected individual II.1, mother of the index-patient, carries the same chromosomal aberration as her daughter, III.1. The individual II.2, father of III.1, does present a normal male karyotype. We were not able to test the karyotype of I.1 and I.2, grandparents of the index patient, due to their missing compliance; nevertheless, they are healthy individuals without any clinical findings resembling the syndromic features of II.1 and III.1.

Given the similar clinical and genetic findings of both patients the chromosomal aberration occurred de novo in patient II.1 at embryonic stage and was transmitted to patient III.1.

## Conclusions

To our knowledge, our related patients are the unique two described individuals with combined features from two independent syndromes. CANPMR syndrome (OMIM 614756) is a rare genetic disorder of the nervous system presenting with cerebellar ataxia and mild to severe mental retardation, whose genetic cause was recently described to be *CAMTA1* haploinsufficiency [15]. Chromosome 1p32-p31 deletion syndrome (OMIM 613735) presents with hypoplasia/aplasia of the corpus callosum, one of the most common congenital abnormalities of the CNS [27], accompanied by ventriculomegaly with or without hydrocephalus and developmental delay. The haploinsufficiency of *NFIA* was implied by Lu et al [17] to be the molecular cause of this syndrome.

According to the last insights on the genetic patho-physiology of these two syndromes [15, 17], the complex disorder affecting our two related patients is very likely caused by the simultaneous haplo-insufficiencies of *NFIA* and *CAMTA1*, which are the breakpoints of a paracentric inversion in the short arm of chromosome 1. The inversion-associated disruption of *NFIA*’s and *CAMTA1*’s reading frames causes very likely the decay of the remaining mRNA strains (corresponding to *NFIA* exons 1–2 and, respectively *CAMTA1* exons 1–4), which are transcribed from the affected alleles of both genes.

**Fig. 5:**
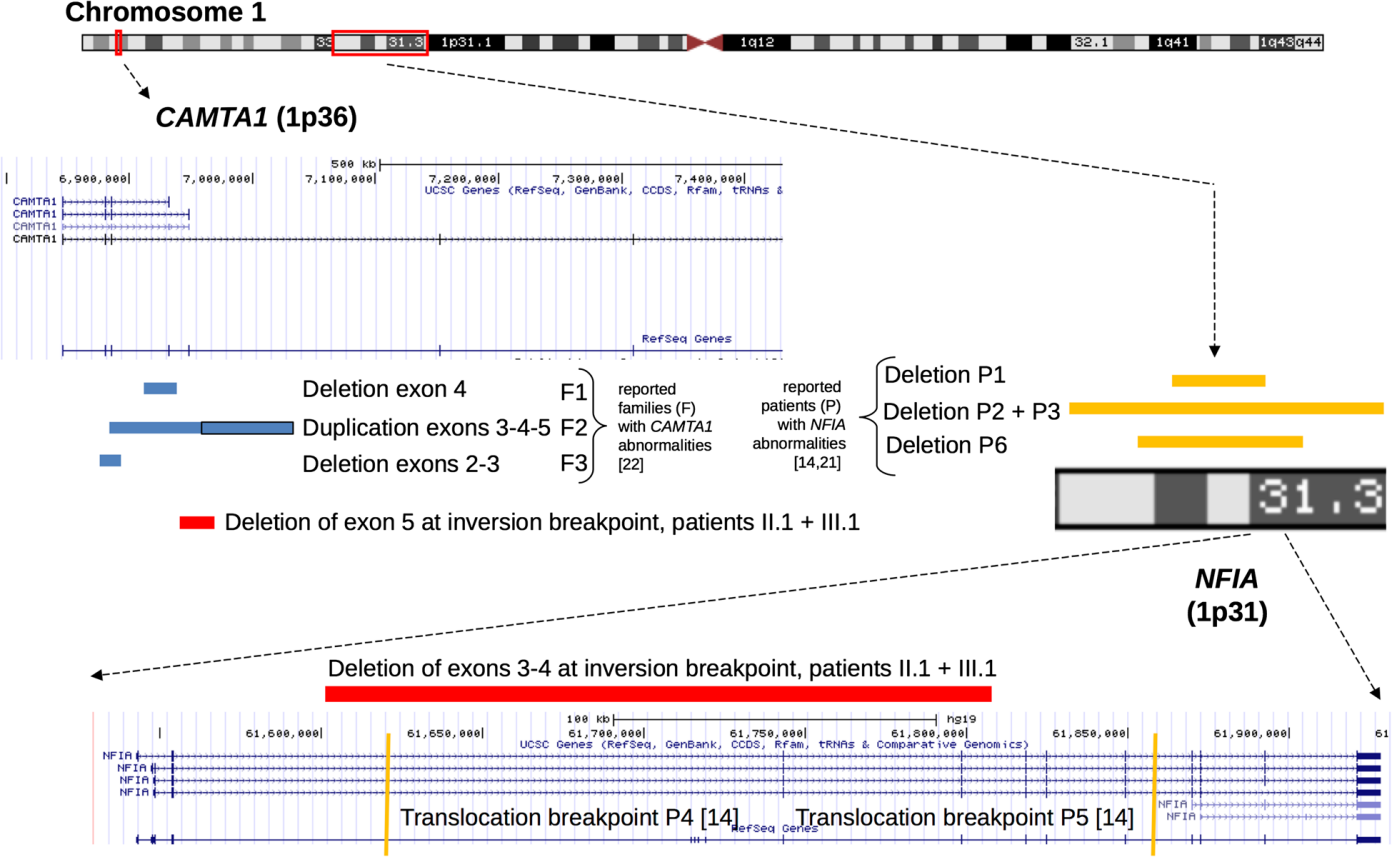
Schematic representation of the two affected genes (*CAMTA1* and *NFIA*) in our two related patients (II.1 and III.1) and in other previously reported families and patients. The blue bars (for *CAMTA1*) and the orange bars and lines (for *NFIA*) represent deletions, duplications and translation breakpoints affecting the two genes in other reported families (F) and single patients (P). The red bars indicate the two simultaneous deletions disrupting *CAMTA1* (1p36.31) and *NFIA* (1p31.3), which affect our two patients (II.1 and III.1) (scheme based on UCSC genomic bioinformatics, GRC37/hg19)

If a truncated NFIA protein is synthesized, the C-terminal transactivation and repression domains would completely lack and therefore the truncated NFIA protein would be unable to exert his effector function. The absence of seizures in our two patients may exclude that *NFIA* haplo-insufficiency causes epileptic seizures, which were described in 3 out of 5 patients reported by Lu et al. On the contrary, the urinary tract abnormalities and VUR in 3 out of 5 patients from Lu et al and in our 2 patients (carrying mono-allelic *NFIA* disruption) seem to reinforce the view that NFIA plays a major role in the human urinary tract development. Remarkably, our patients carry mono-allelic disruptions of only two genes in comparison with the patients reported by Lu et al. [17] and Koehler et al. [25], which harbored larger deletions encompassing several other genes apart from *NFIA* (Fig. 5). This fortuitous genetic condition strengthens the casual association between *NFIA* haplo-insufficiency and some clinical findings described in our study.

The pathogenic insights on *CAMTA1* haploinsufficiency are enforced by the clinical findings of our two patients. Ataxic and delayed gait, dysmetric movements, instability, macrocephaly and ventriculomegaly, cerebellar and cortical abnormalities at MR scans strengthen the clinical stigmata of CANPMR syndrome. While all former patients reported by Thevenon et al carried intragenic deletions and duplications disrupting the N-terminal CG-1 domain (short deletion of exon 4 in familiy 1, duplication of exons 3 to 5 in family 2, short deletion of exon 3 in familiy 3), the chromosomal inversion harbored by our 2 patients represents the first report on mono-allelic deletion of C-terminal domains TIG, IQ motif and Ankirin from *CAMTA1* reading frame and shows the patho-physiological consequence of this genetic event. Among 11 patients from 3 families reported by Thevenon et al, 9 out of 11 patients are reported to have a pathologic intelligence quotient (IQ) between 40 and 67 and a mild to severe intellectual disability (ID), like our 2 patients.

Although an inversion-dependent imbalance on the expression of neighboring genes, located nearby to inversion breakpoints, cannot be theoretically excluded as co-factor responsible for the clinical phenotype, such positional effect seems to be very unlikely in our cases. In fact, no gene mapping in the proximity of deletion 1p36.31 (e.g. *DNAJC11, THAP3, PHF13, VAMP3, PER3, UTS2*) or in the proximity of the deletion 1p31.3 (e.g. *CYP2J2, HOOK1, FGGY, TM2D1, INADL, KANK*) was associated with brain and urinary tract abnormalities to date.

Taken together, in our two patients the study of the mono-allelic and simultaneous disruption of genes *CAMTA1* and *NFIA*, whose haploinsufficiencies were already associated to two independent pathological phenotypes [15, 17], sheds further light on the clinical and genetic features of the rare developmental disorders CANPMR syndrome and chromosome 1p32-p31 deletion syndrome.

## Methods

Upon extraction of DNA from total peripheral blood leucocytes, conventional karyotyping was performed using G-banding techniques on stimulated blood lymphocytes with standard cytogenetic methods and analyzed at 500–550 band resolution. Karyotypes were described according to the International System for Human Cytogenetic Nomenclature (ISCN 2013).

SNP array was performed using an Infinium CytoSNP-850 K microarray (Illumina, San Diego, CA, USA) with an average resolution of 18Kb and a practical resolution in genes *CAMTA1* and *NFIA* of 1 Kb according to the manufacturer’s protocol. The data analysis was done using BlueFuse V4.2 software. The gene alignment was done using the University of California Santa Cruz (UCSC) genomic bioinformatics browser. The two deleted regions were mapped using the Genome Research Consortium Build 37 human/human genome 19 (GRCh37/hg19). The GenBank, Ensembl and OMIM browser accession numbers for *CAMTA1* are NM_015215, ENST00000303635, ENSG00000171735, MIM 611501 and for *NFIA* are NM_001134673, ENST00000403491, ENSG 00000162599, MIM 600727.

Cerebral MRI was performed on clinical 1.5 T MRI systems (Magnetom Avanto and Aera, Siemens Medical, Germany) using standardized MRI protocols including multiplanar T1 and T2-weighted MR-sequences.

### Patient consent

The authors obtained the patient consent for the publication of the data.

## Abbreviations

CANPMR: cerebellar ataxia non progressive with mental retardation
SARA: scale for the assessment and rating of ataxia
SON-R: Snijders-Oomen non-verbal test
