# Correction to: CANPMR syndrome and chromosome 1p32-p31 deletion syndrome coexist in two related individuals affected by simultaneous haplo-insufficiency of *CAMTA1* and *NFIA* genes

**DOI:** 10.1186/s13039-021-00534-5

**Published:** 2021-03-09

**Authors:** Emanuele G. Coci, Udo Koehler, Thomas Liehr, Armin Stelzner, Christian Fink, Hendrik Langen, Joachim Riedel

**Affiliations:** 1 Center of Social Pediatrics and Pediatric Neurology, General Hospital of Celle, 29221 Celle, Germany; 2 Medizinisch Genetisches Zentrum, 80335 Munich, Germany; 3 Institute of Human Genetics, Friedrich Schiller University, Jena University Hospital, 07743 Jena, Germany; 4 Department of Radiology, General Hospital of Celle, 29223 Celle, Germany

## Correction to: Molecular Cytogenetics (2016) 9:10 10.1186/s13039-016-0219-y

Following publication of the original article [1], an error was identified in the article title: the gene name *NFIA* was incorrectly captured as *NIFA*. The article title has been updated above and in the original article.
